# How the James Webb Space Telescope Is Revealing the Weather and Chemistry on Planets around Other Stars

**DOI:** 10.1021/acscentsci.4c00820

**Published:** 2024-06-20

**Authors:** Andy Extance

The weather on the gas giant Ditsö̀ is literally alien. Clouds of solid quartz appear and disappear thanks to silicon and oxygen atoms continually evaporating from and then condensing in its atmosphere.

**Figure:**
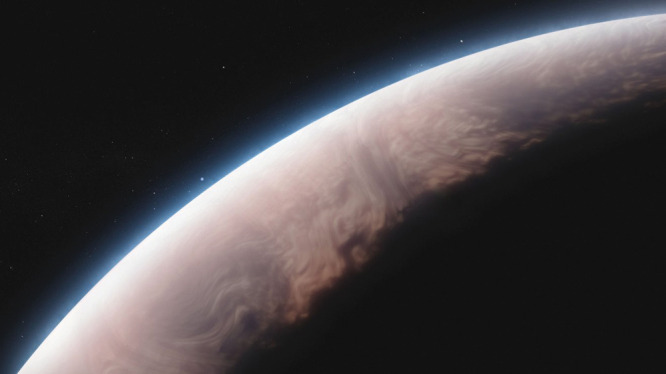
Clouds of quartz nanocrystals on the gas giant exoplanet Ditsö̀, also known as WASP-17 b, continually appear and disappear. Depicted is an artist’s concept of Ditsö̀. Credit: NASA, ESA, CSA, and R. Crawford (STScI).

Ditsö̀, also known as WASP-17 b, orbits a star 1,300 light-years from Earth. One side of the planet is perpetually locked so that it faces the star and is thus permanently illuminated as the dayside. That half of the planet reaches temperatures of 1,773 K (1,500 °C), according to Hannah Wakeford, an astrophysicist at the University of Bristol.

The nanocrystals in Ditsö̀’s clouds heat up and break apart as they travel through the dayside but begin to cool down as they approach the planet’s darker half. “You’ve got this tinkling of crystals forming and shrouding the nightside,” Wakeford says.

Wakeford knows this as coleader of one of the first teams of scientists to use infrared (IR) spectrometers on board the $10 billion James Webb Space Telescope (JWST), launched in December 2021. The JWST is the world’s latest flagship satellite-based telescope, its spectrometers recording the brightness of IR-wavelength light invisible to human eyes. Scientists want to record light from stars—specifically, the colors whose brightness is reduced when the planets that orbit those stars pass across them. Those dimmer colors have been absorbed by chemicals in the planets’ atmospheres.

The researchers can then determine what those chemicals are by interpreting the wavelengths absorbed, giving an unprecedented view of chemistry elsewhere in our galaxy.

The JWST is the largest space telescope ever launched, with a 6.5 m mirror made of 18 hexagonal, gold-coated beryllium sections collecting and focusing light. That’s nearly three times as wide as the mirror in its predecessor, the Hubble Space Telescope. The mirror helps make the JWST 100 times as sensitive as Hubble, which allows it to pick up much dimmer light.

The JWST observations that revealed Ditsö̀’s strange cloud formations were part of a series of experiments done between July and December 2022. Those experiments are still revealing chemical processes and bizarre weather on exoplanets—planets that lie outside our solar system, many hundreds of light-years away. They are also continuing to help astrophysicists decide how to use the JWST to answer fundamental questions about what the rest of the universe looks like, including whether our scientific knowledge holds true there.

“These are very different kinds of worlds that we’re talking about and trying to understand,” Wakeford says. They often have harsh environments that could enable phenomena we have never seen. “These planets are our laboratories to understand how we can take all of the physics and chemistry we think we understand to the extremes,” Wakeford says.

## Inventing ways to study exoplanet chemistry

The JWST’s original aim was to search for the first galaxies to form after the big bang, says the University of Arizona’s Marcia Rieke, an astronomer who led the design of the telescope’s Near Infrared Camera (NIRCam). The instrument detects two colors of IR light from the same piece of sky: shorter wavelengths of 600–2,300 nm, which are closer to those of visible light, and longer wavelengths of 2,400–5,000 nm.

Rieke’s team members hoped that by measuring within those ranges, they would detect light from very distant galaxies. That light has traveled so far that it was emitted not long after the big bang. Measuring it would help us understand how the universe formed.

The team realized that the longer wavelengths would also be good for recording spectra of “interesting molecules like water, carbon dioxide, and methane,” Rieke says. For example, ice can absorb light at around 3,000 nm. Being able to detect light of longer wavelengths is useful for researchers seeking to detect chemicals, such as ice, floating freely in galaxies. They can look at the changes in spectra as those chemicals pass in front of the light source.

One of the team members, NASA astrophysicist Thomas Greene, then suggested that they use NIRCam to study light absorbed by planets as they travel past their stars for clues about the chemical makeup of the planets’ atmospheres.

For researchers to study the light absorbed by an atmosphere, the JWST’s instruments first measure the brightness and color of a star’s light in high precision. The instruments then measure the same information from that star when one of its planets is traveling across it, at which time chemicals in the planet’s atmosphere absorb some photons of light.

Subtracting the second measurement from the first leaves a spectrum of different colors of light absorbed by the planet’s atmosphere. From the exact wavelengths of light absorbed, astroscientists can deduce what chemicals are present in the atmosphere.

Researchers have already used this method to carry out an important task: confirming methane’s presence in an exoplanet’s atmosphere. No satellite telescope before the JWST had been able to find any methane on exoplanets—even though chemical simulations predict that exoplanets at temperatures below 1,000 K should have more methane than any other carbon-containing compound. A few ground-based telescopes had found weak signs on a planet called Wadirum, also called WASP-80 b, but they were too weak to count as a definite observation. Orbiting a star approximately 162 light-years from Earth, Wadirum is about the size of Jupiter but about half the mass. Its dayside temperature is 851 K.

**Figure:**
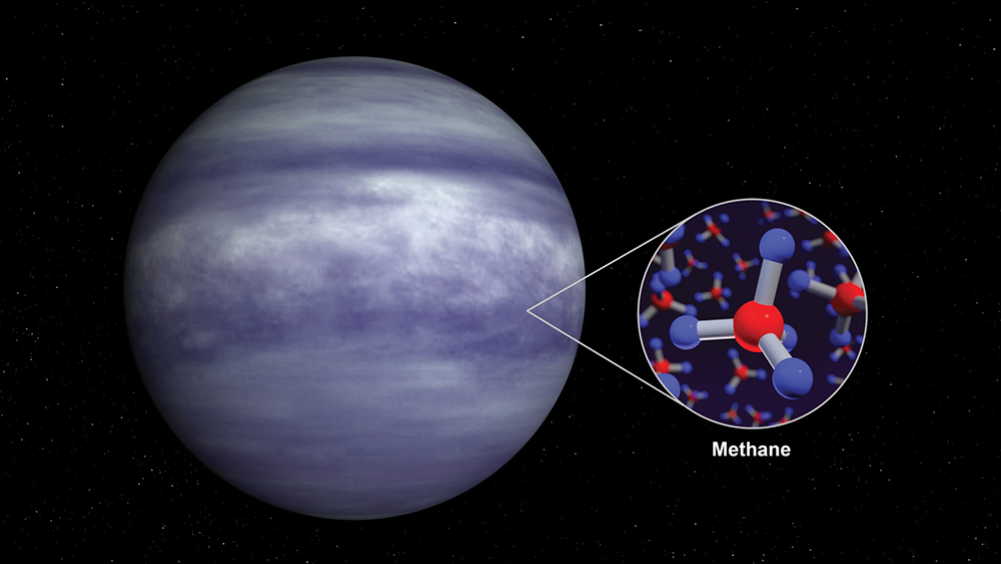
An artist’s rendering of the exoplanet Wadirum, or WASP-80 b. It is the first planet on which a space telescope has detected methane, an important achievement for the James Webb Space Telescope. Credit: NASA.

In November 2023, a team including Greene and Rieke reported that it had confirmed the gas on Wadirum. NIRCam made the group’s analysis possible because it detects wavelengths that methane strongly absorbs. Ground-based telescopes detect other wavelengths, but methane absorbs those relatively weakly.

Greene says methane still seems less abundant on exoplanets than previous simulations would lead astroscientists to expect. But he points out that hotter, less dense layers of gas on Wadirum end up higher in the atmosphere, where it’s easiest for the JWST to measure. Methane may be lurking in the lower, denser layers, where it’s harder to find.

Greene notes that Hubble could record some infrared spectra, primarily of water. That has helped it study 70 exoplanets so far in its 34 years in orbit. But the much more sensitive JWST is already far outpacing its ancestor. “Webb has observed close to 70 already, and it’s going to do more by the end of this year,” he says.

Wakeford is excited about improving our understanding of exoplanet carbon chemistry from the spectra of molecules in their atmospheres. This “is a really important part of learning about how these planets formed and evolved through time,” she says.

Carbon chemistry data help test predictions about how a planet’s chemistry changes through its life. To make those predictions, exoplanet scientists have exploited another of their key tools: computer models.

## Models matter

Ditsö̀’s alien yet beautiful-sounding quartz clouds first showed up as a mysterious infrared absorption. It was in the new range of IR colors detected by the JWST’s mid-infrared instrument (MIRI), which covers light wavelengths of 5,000–28,000 nm.

MIRI enabled the quartz detection, which Wakeford calls “something completely new.” The observations were so surprising that the JWST data weren’t confirming expectations predicted by computer models. The researchers had to instead work in reverse and use models to explain reality.

The quartz clouds are “a very big highlight,” says Sarah Moran, a planetary scientist at the University of Arizona. Scientists had previously inferred the presence of clouds on exoplanets by detecting gases. This is the “first detection by actually seeing the molecular bonds from a cloud itself,” she says.

Though Moran didn’t work on the quartz cloud study, she often collaborates with its authors on JWST projects. Her work with them involves using both modeling and earthbound experiments to help understand what spectra from the JWST tell us about exoplanet weather.

**Figure:**
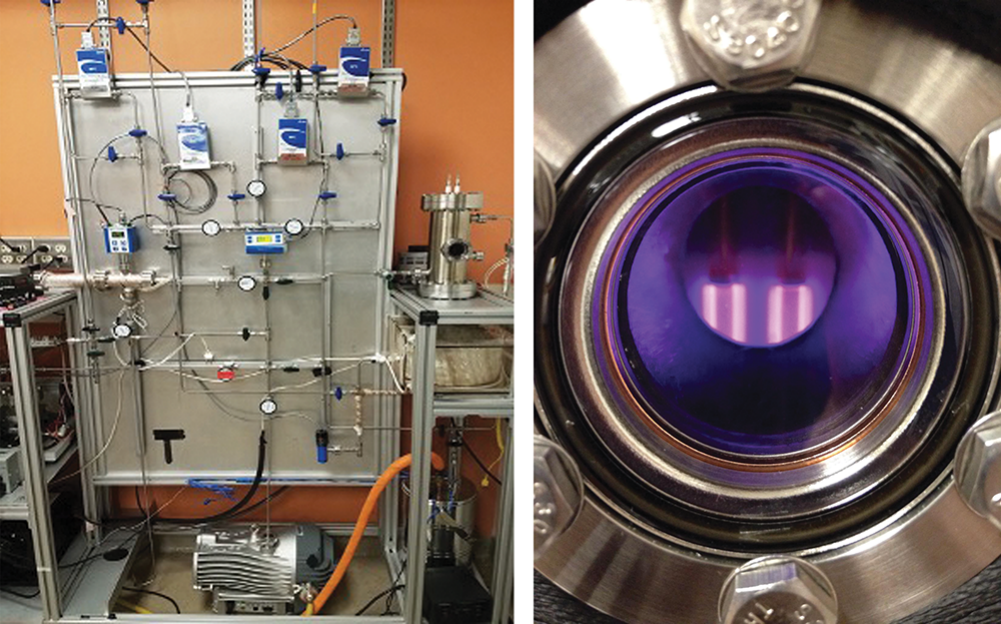
Sarah Moran and colleagues flow different gases that may represent exoplanet atmospheres into the chamber on the table on the right hand side of the left image. The scientists expose the gases in that chamber to the plasma energy source shown in the right image. The plasma mimics energy in the form of radiation from the exoplanet's star entering that atmosphere. That energy initiates chemical reactions and starts to make fog. The researchers can test the smog for its components and see how it might look to telescopes. Credit: Sarah Moran/Johns Hopkins University (left), Sarah Hörst/Johns Hopkins University (right).

The experiments involve flowing gases that may represent an exoplanet’s atmosphere, such as hydrogen, methane, ammonia, carbon monoxide, and nitrogen, into a chamber about the size of a liter bottle. “Then we expose that chamber to an energy source supposed to mimic starlight coming into that atmosphere,” which initiates chemical reactions in the mixture, Moran says. “We can test that for what it’s made of and how that might look to telescopes.”

Moran also computationally models particles in the atmosphere to help interpret exoplanet absorption data from the JWST. She calculates the temperatures and pressures of different layers in the planet’s atmosphere using data from various telescopes. Once that’s done, “we can think about what the chemistry is like,” she says. That typically involves fine-tuning the composition of elements or the conditions of the atmosphere. Tweaks include adding more carbon and oxygen or factoring things like clouds and smog into the models.

“We create atmospheric structures and compositions and compare the expected observations to reality,” says the National Astronomical Observatory of Japan’s Kasumasa Ohno, who also models exoplanet atmospheres.

Like with Ditsö̀’s quartz clouds, the reality of the JWST’s observations of Bocaprins, also known as WASP-39 b, challenged the modeling skills of Ohno and his colleagues. A Jupiter-sized exoplanet 698 light-years from Earth, Boca-prins orbits very close to its star, Malmok, or WASP-39—just one-fifth the distance between Mercury and the sun—making it very hot.

**Figure:**
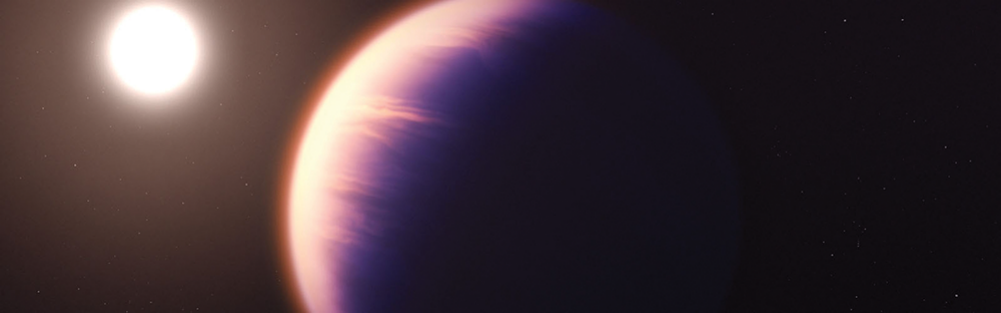
An illustration of Bocaprins, also known as WASP-39 b. The James Webb Space Telescope detected sulfur dioxide forming in its atmosphere—the first starlight-driven reaction found on an exoplanet. Credit: NASA, ESA, CSA, Joseph Olmsted (STScI).

Hubble had found Bocaprins to have a water-rich atmosphere, and it was the first exoplanet studied by the JWST in July 2022. Ohno and colleagues analyzed the elements floating around it and were able to identify carbon dioxide in its atmosphere. It was the first time the gas had been detected on an exoplanet.

But there was something else initially unexpected and unidentified in the data, recalls Wakeford, who co-led the whole group studying Bocaprins. “We had about 30 chemists/physicists in the team that were trying to work out what was going on,” she says. Using computational models, they found that the mystery data arose from sulfur dioxide, produced by starlight-driven reactions between hydrogen sulfide and water. This was the first photochemical product detected on an exoplanet.

In this way, modeling helps the JWST reveal what happens in the wildly alien conditions on exoplanets. Astroscientists often need to call on simulations to explain their new data rather than have data confirm predictions.

## No Planet B

Anyone feeling alone in the universe might be more interested in smaller, rockier planets such as Earth than in gas giants like Ditsö̀, Wadirum, and Bocaprins. Scientists have until now mainly studied larger exoplanets, because they are easier to see and more common. But the sensitivity of the JWST’s instruments should also help investigations into planets of a similar size to our own.

A first step came in studying the TRAPPIST-1 system, an effort Greene led. The star at the system’s center is “really puny,” he says, about a 10th the size of our sun. It is surrounded by seven rocky planets, all of which the JWST can watch as they cross the star.

When the telescope observed the Earth-sized TRAPPIST-1 b and TRAPPIST-1 c planets, scientists expected their daysides to emit infrared light moderately brightly. “But no, both are rather bright, to the point where it looks like it’s hot rock,” which suggests that neither planet has any significant atmosphere, Greene says. “We’re seeing one face illuminated by the star. It’s so hot, it looks like there’s not much radiation from the star being spread around to the other side either. That’s another indicator of no atmosphere.”

**Figure:**
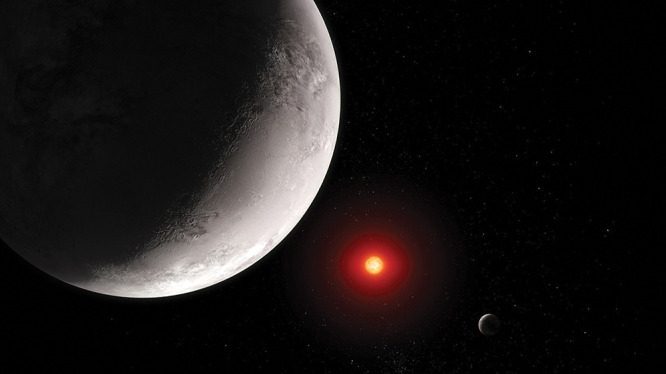
The way the planet TRAPPIST-1 c (artist’s concept shown) absorbs heat from its “puny” star suggests that it has no atmosphere. Credit: NASA, ESA, CSA, Joseph Olmsted (STScI).

Greene notes that the conditions that led to life on Earth are extraordinary and that planets can meet many other fates. “I would be surprised if many planets had atmospheres very similar to Earth’s today,” he says.

Earth’s small size and atmospheric composition aren’t its only unusual characteristics. Wakeford points out that “across the entire range of detected exoplanets, the solar system is a very, very cold place.”

“Every single planet that we have enough information [about] from the JWST is likely uninhabitable,” Wakeford says. As such, one lesson from studies of other planets would be to take care of our own.

In fact, scientifically, there’s no reason to expect an exoplanet to be like Earth. Exoplanets will all be diverse and unique, meaning finding a planet like ours isn’t the motivation for Wakeford. “There’s this wonderfully egotistical drive towards ‘Where’s the next Earth?’ ” she says. “But we can find out so much about these very different environments from just the photons we get. That is, to me, really just a joyful thing.”

*Andy Extance is a freelance contributor to*Chemical & Engineering News*, the independent news outlet of the American Chemical Society.*

